# Predicting Tumor Budding Status in Cervical Cancer Using MRI Radiomics: Linking Imaging Biomarkers to Histologic Characteristics

**DOI:** 10.3390/cancers13205140

**Published:** 2021-10-14

**Authors:** Gun Oh Chong, Shin-Hyung Park, Nora Jee-Young Park, Bong Kyung Bae, Yoon Hee Lee, Shin Young Jeong, Jae-Chul Kim, Ji Young Park, Yu Ando, Hyung Soo Han

**Affiliations:** 1 Department of Obstetrics and Gynecology, School of Medicine, Kyungpook National University, Daegu 41944, Korea; gochong@knu.ac.kr (G.O.C.); mylyh@naver.com (Y.H.L.); 2 Department of Obstetrics and Gynecology, Kyungpook National University Chilgok Hospital, Daegu 41944, Korea; 3 Clinical Omics Research Center, School of Medicine, Kyungpook National University, Daegu 41944, Korea; pathpjy@naver.com (N.J.-Y.P.); hshan@knu.ac.kr (H.S.H.); 4 Department of Radiation Oncology, School of Medicine, Kyungpook National University, Daegu 41944, Korea; bae1675@naver.com (B.K.B.); jckim@knu.ac.kr (J.-C.K.); 5 Cardiovascular Research Institute, School of Medicine, Kyungpook National University, Daegu 41944, Korea; doyu94@daum.net; 6 Department of Pathology, School of Medicine, Kyungpook National University, Daegu 41944, Korea; jyparkmd@knu.ac.kr; 7 Department of Nuclear Medicine, School of Medicine, Kyungpook National University, Daegu 41944, Korea; syjeong@knu.ac.kr; 8 Department of Physiology, School of Medicine, Kyungpook National University, Daegu 41944, Korea

**Keywords:** radiomics, cervical cancer, magnetic resonance imaging, tumor budding

## Abstract

**Simple Summary:**

Tumor budding is a histopathologic characteristic which has led to a growing interest in the prognosis prediction of cancers of various sites. We aimed to evaluate whether imaging biomarkers could predict tumor budding status. Preoperative MRI radiomic features were used as imaging biomarkers. Four machine learning classifiers were applied to build prediction models using a training dataset. Internal validation was performed to validate the built models. As a result, radiomics-based models predicted tumor budding status with a mean area under the receiver operating characteristic value of 0.816 and a mean accuracy of 0.779 in the independent test dataset. Final selected features were mostly from filtered images, implying the importance of filtering methods in radiomics. Preoperative prediction of tumor budding status may help personalize treatment in cervical cancer patients.

**Abstract:**

Background: Our previous study demonstrated that tumor budding (TB) status was associated with inferior overall survival in cervical cancer. The purpose of this study is to evaluate whether radiomic features can predict TB status in cervical cancer patients. Methods: Seventy-four patients with cervical cancer who underwent preoperative MRI and radical hysterectomy from 2011 to 2015 at our institution were enrolled. The patients were randomly allocated to the training dataset (*n* = 48) and test dataset (*n* = 26). Tumors were segmented on axial gadolinium-enhanced T1- and T2-weighted images. A total of 2074 radiomic features were extracted. Four machine learning classifiers, including logistic regression (LR), random forest (RF), support vector machine (SVM), and neural network (NN), were used. The trained models were validated on the test dataset. Results: Twenty radiomic features were selected; all were features from filtered-images and 85% were texture-related features. The area under the curve values and accuracy of the models by LR, RF, SVM and NN were 0.742 and 0.769, 0.782 and 0.731, 0.849 and 0.885, and 0.891 and 0.731, respectively, in the test dataset. Conclusion: MRI-based radiomic features could predict TB status in patients with cervical cancer.

## 1. Introduction

Precision medicine refers to medicine optimized to the genotypic and phenotypic characteristics of an individual and disease. The growing focus on precision medicine in oncologic fields is leading to an increased demand for predictable biomarkers, which can be used in decision making in clinical practice. High-throughput data mining from medical imaging, also called radiomic analysis, enables the development of an imaging biomarker with the aid of recent advances in computer science. The term “radio” in radiomics means radiology, and “omics” originally refers to the comprehensive assessment of various molecule types within the cells of an organism. Omics includes various research fields, including genomics, transcriptomics, proteomics, phenomics and radiomics. Because such omics fields can interact with each other in the body, it is necessary to find an association between the different research fields in order to comprehensively improve the understanding of tumor biology and their clinical behavior.

Tumor budding (TB) is defined as the presence of a single cancer cell or clusters of up to four cancer cells at the invasive tumor front or within the main tumor body. It has emerged as a new possible biomarker to predict unfavorable clinical outcomes in various cancer types from different organs [1,2], including colorectal, esophageal, pancreatic, and cervical cancers [3]. Our previous studies demonstrated that TB status was associated with tumor recurrence, lymph node metastasis, and poor overall survival in cervical cancer, suggesting that it may be a prognostic biomarker [4,5].

Nonetheless, one of the disadvantages of histopathologic prognostic factors is that we cannot determine them before surgical resection. It is conceivable that the treatment strategy may vary according to each patient’s given prognosis (e.g., neoadjuvant therapy/surgical extent) if the pathologic prognostic factors, such as TB status, can be predicted reliably by a preoperative imaging study. Thus, we evaluated the possibility of magnetic resonance imaging (MRI) radiomic features to predict TB status in patients with cervical cancer who were undergoing radical hysterectomy.

## 2. Materials and Methods

### 2.1. Study Population

The records of 136 patients with cervical cancer who underwent preoperative MRI and radical hysterectomy from 2011 to 2015 were reviewed retrospectively. This study was conducted in accordance with the guidelines and approval from the institutional review board of Kyungpook National University Hospital and Kyungpook National University Chilgok Hospital. The institutional review board provided a waiver of consent. The patients were included based on the following criteria: (a) pathologically confirmed cervical cancer; (b) evaluated by MRI preoperatively; and (c) pathologically described TB status. The exclusion criteria were as follows: (a) not evaluated by MRI preoperatively; (b) neoadjuvant chemotherapy before surgery; (c) no definite cervical tumor on preoperative MRI; (d) did not have contrast-enhanced T1-weighted imaging or T2-weighted imaging; and (e) inadequate image quality due to an intractable artifact. Eventually, 76 patients were enrolled in this study (Figure S1). The enrolled patients were randomly allocated to the training dataset (*n* = 48) and test dataset (*n* = 26).

### 2.2. Histopathological Evaluation

We reviewed the clinicopathologic information from the archives of medical records and corresponding hematoxylin and eosin (H&E)-stained slides of cervical cancers. The clinicopathologic parameters included age, FIGO stage, primary tumor size, histologic subtype, lymphovascular invasion (LVI), deep stromal invasion, parametrial invasion, lymph node status, and TB status. For each case, all available H&E slides were independently reviewed for histopathological features and the quantitative assessment of TB was performed by two pathologists (N.J.P and J.Y.P). The number of reviewed slides ranged from 8 to 25. TB was defined as an isolated single cancer cell or small cell clusters comprising ≤4 tumor cells located within the tumor area, with reference to the examination method used in our previous paper [4]. In brief, the greatest degree of TB was selected using the medium power field (10× objective, Olympus, BX-53), and the highest number of TB per high-power field (20× objective) was determined (the so-called “hotspot” counting method).

### 2.3. Image Acquisition

The preoperative MRI was obtained with three MR scanners (Discovery MR750, GE Healthcare, 3T; Signa Excite, GE Healthcare, 1.5T; Magnetom Avanto, Siemens Healthcare, 1.5T). The same MR imaging sequences were obtained from all patients, including axial and sagittal T2-weighted fast spin-echo (FSE), axial T1-weighted FSE, and axial and sagittal T1-weighted FSE with fat saturation after gadodiamide administration. The MRI protocol was as follows: axial T2-weighted images (repetition time/echo time, 3500–4500/90–110; slice thickness, 5 mm, no gap; field of view, 22 × 22 cm to 26 × 26 cm; matrix, 320 × 224, 384 × 256), sagittal T2-weighted images (repetition time/echo time, 4000–6000/90–110; slice thickness, 5 mm, no gap; field of view, 24 × 24 cm; matrix, 384 × 256, 416 × 256), and axial T1-weighted images (repetition time/echo time, 700–800/minimum; slice thickness, 5 mm, no gap; field of view, 22 × 22 cm to 26 × 26 cm; matrix, 320 × 256, 384 × 224).

### 2.4. Segmentation and Preprocessing

**Figure 1:**
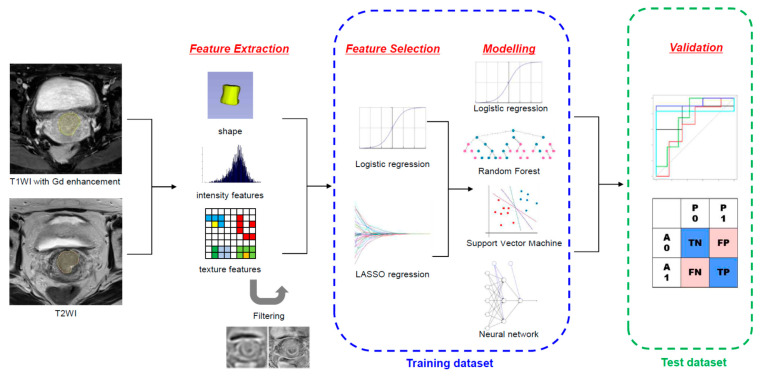
Illustration of the overall process of MRI radiomic analysis to predict tumor budding status.

Figure 1 illustrates the schematic diagram of the radiomic analysis. Before image segmentation, the patient-sensitive information was anonymized. The primary tumor lesion was semi-manually segmented on axial gadolinium-enhanced T1-weighted images and T2-weighted images by the two radiation oncologists (S.H.P. and B.K.B), using the annotation tool of 3D Slicer version 4.11.0 (www.slicer.org (accessed on 26 June 2020)).

The MRI images were resampled with a pixel space of 0.8 and slice thickness of 5 mm using a linear interpolation algorithm. Because the MRI signal intensities are relative values, we normalized the intensity using PyRadiomics. Normalization was based on the all-gray values contained within the image, not just those defined by the region of interest (ROI). The normalization scaling factor was set to 100. All voxel values were shifted by 300 to ensure that most voxels had positive values.

### 2.5. Radiomic Feature Extraction and Selection

The radiomic features were extracted from the ROIs on post-contrast T1-weighted and T2-weighted images using Pyradiomics version 3.0 [6]. Eighteen first-order features, 14 shapes, 24 gray-level co-occurrence matrix, 16 gray-level size zone matrix, 16 gray-level run length matrix, 5 neighboring gray tone difference matrix, and 14 gray-level dependence matrix features were extracted from the original and filter-applied images from postcontrast T1-weighted and T2-weighted images, resulting in 2074 total features. The filters included Laplacian of Gaussian filters with sigma values of 1.0 and 3.0 mm and wavelet filters (eight combinations of high- and low-pass filters on each dimension). The detailed definition of each feature has been described elsewhere (https://pyradiomics.readthedocs.io/en/latest/index.html (accessed on 10 October 2021)). A fixed bin width of 5 was used. The Image Biomarkers Standardization Initiative guideline was followed [7]. Each feature was standardized using z-score normalization to have a mean of 0 and a standard deviation (SD) of 1 [8].

The features to build the prediction models were selected using the training dataset. In the first step, a logistic regression (LR) was used to screen the potential features. Only the features with *p* < 0.05 were chosen to proceed to the next selection step. In the second step, the least absolute shrinkage and selection operator (LASSO) regression was employed to select the features to construct a prediction model with a five-fold cross validation.

### 2.6. Statistical Analysis and Machine Learning (ML) Model Building

The differences in patient characteristics between the training and test dataset were compared using Student’s *t*-test, Pearson chi-square test, and Fisher’s exact test. Four ML classifiers, including LR, random forest (RF), support vector machine (SVM), and neural network (NN), were used to build a model to predict TB status in the training set. We implemented the R package of “neuralnet” (version 1.44.2) which trains NN models using backpropagation, resilient backpropagation, or the globally convergent algorithm based on resilient backpropagation [8,9]. In our neural network model, resilient backpropagation was applied. During classifier training, the hyperparameters were optimized by grid search. Then, the trained ML models were validated on the test dataset. The predicted likelihood was calculated using the area under the receiver-operating characteristic (ROC) curve to measure the ML models’ performance. Moreover, the accuracy, sensitivity, specificity, positive predictive value, and negative predictive value were assessed. All statistical analyses, including ML, were performed using R version 3.2.4 (R Foundation for Statistical Computing, Vienna, Austria). *p* < 0.05 was considered to indicate a statistically significant difference. The R package of “glmnet,” “caret,” “randomForest,” “kernlab,” and “neuralnet,” were used for analysis. The code implemented in this work is available at http://github.com/RO-KNU/tumorbudding/ (accessed on 25 September 2021).

## 3. Results

Seventy-four patients were enrolled in this study (Figure S1). The median age of patients at diagnosis was 50 years (range, 28–78). Fifty-four patients (73.0%) had squamous cell carcinoma, fifteen (20.3%) had adenocarcinoma, and five (6.8%) had adenosquamous cell carcinoma. The International Federation of Gynecology and Obstetrics (FIGO) stage was IB1 in 14 patients (18.9%), IB2 in 24 (32.4%) patients, IB3 in 4 patients (5.4%), IIA in 1 patient (1.4%), IIB in 8 patients (10.8%), and IIIC1 in 23 patients (31.1%). The median tumor size was 2.5 cm (range, 0.4–8.0). The median TB count was 4.0 (0–40). The TB status was binarily classified with a cutoff value of 4.

The patients were semi-randomly partitioned into a training set of 48 patients and a test set of 26 patients (Figure S1). The patients from the training set and those from the test set were not significantly different in terms of TB counts (*p* > 0.999), age (*p* = 0.400), FIGO stage (*p* = 0.517), histology (*p* = 0.580), tumor size (*p* = 0.417), LVI (*p* = 0.828), deep stromal invasion (*p* = 0.903), parametrial invasion (*p* > 0.999), and lymph node metastasis status (*p* = 0.760) (Table 1).

In a univariate logistic regression analysis, 29 features were significantly associated with TB status in the training dataset. The selected features from the logistic analysis were subjected to the next feature selection step by LASSO regression (Figure S2). Among them, 20 features were finally selected to build prediction models (Table 2). All of these were extracted from filter-applied images (3 with a Laplacian of Gaussian filter and 17 with a wavelet filter). Seventeen features (85%) were texture-related features.

**Figure 2:**
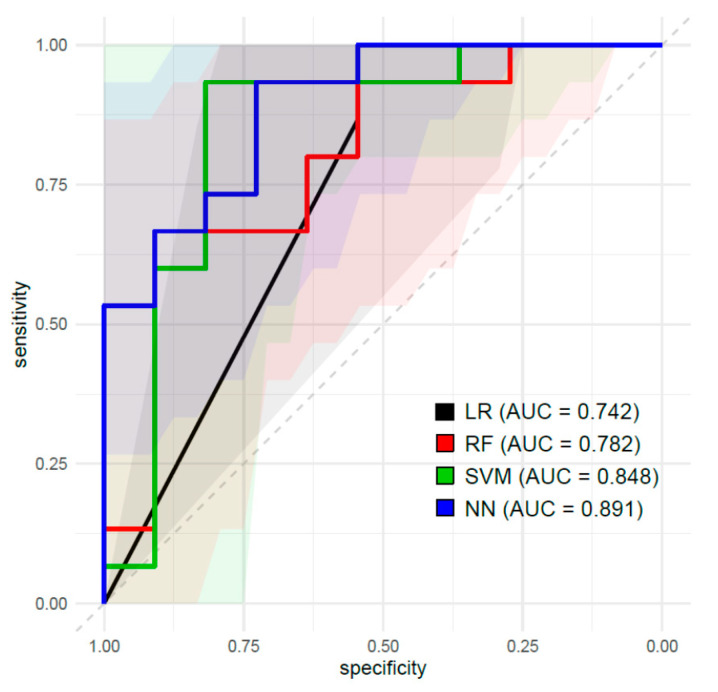
Receiver-operating characteristic (ROC) curves of the prediction models constructed by logistic regression (LR), random forest (RF), support vector machine (SVM), and neural network (NN) algorithms using MRI radiomic features in the test dataset.

Table 3 shows the summary of prediction performance to predict TB status by various ML classifiers in the test dataset. The ROC curves are presented in Figure 2. The AUC values of the models by LR, RF, SVM, and NN were 0.742, 0.782, 0.848 and 0.891, respectively, in the test dataset. The accuracy of LR, RF, SVM, and NN were 0.769, 0.731, 0.885 and 0.731, respectively, in the test dataset.

## 4. Discussion

This study demonstrates that MRI radiomic features could successfully predict TB status in the test dataset. Various ML models with 20 selected radiomic features showed a mean AUC of 0.816 (SD, 0.067) and a mean accuracy of 0.779 (SD, 0.073) in the test dataset. Specifically, AUC values of SVM and NN models were 0.849 (95% CI, 0.740–1.000) and 0.891 (95 CI, 0.768–1.000), respectively. Generally, prediction performance of models with an AUC of 1.0 is perfect, 0.9–0.99 is excellent, 0.8–0.89 is good, 0.7–0.79 is fair, 0.51–0.69 is poor, and 0.5 is no better than when determined by chance [10]. Our SVM and NN models incorporating MRI radiomic features showed good prediction performance in predicting TB status.

Several studies have tried to correlate histopathologic characteristics with radiomic features—in most of which, LVI, one of the well-known adverse prognostic factors, was investigated. In a radiomic nomogram introduced by Li et al. to predict LVI in cervical cancer [11], the authors reported that the prediction model had an AUC, specificity, and sensitivity of 0.727, 0.828, and 0.692, respectively, in the validation dataset. Likewise, a MRI radiomics study by Hua et al. revealed that radiomic features combined with a deep-learning technique in tumors and peritumoral lesions could predict LVI status, with an AUC of 0.775 in the test dataset [12]. However, to the best of our knowledge, no study has investigated the correlation between radiomic features and TB status. This approach to predict histopathologic characteristics using radiomic features is clinically meaningful because histopathologic characteristics can only be confirmed after surgical resection, whereas radiomic features can be determined preoperatively.

**Figure 3:**
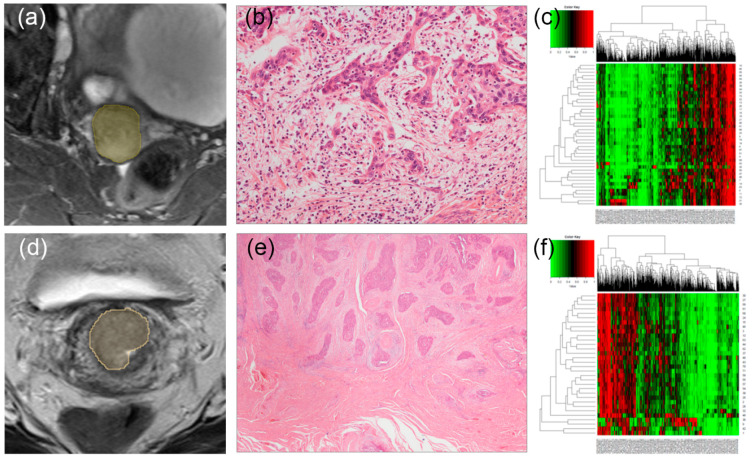
MRI images of two representative cases showing a high tumor budding (TB) count (**a**) and low TB count (**c**). Corresponding hematoxylin- and eosin-stained images at 100× original magnification taken from a radical hysterectomy specimen (**b**,**d**), respectively. (**b**) A patient shows a high TB count. (**d**) A patient shows a low TB count. Corresponding heatmaps showing the radiomic features of those who had a high TB count (**e**) and low TB count (**f**). Each row and column represent one patient and one radiomic feature, respectively.

It has been noted that TB reflects the epithelial–mesenchymal transition (EMT) process [13,14]. EMT is known as a multistep dynamic process of tumor cell dissociation from the main tumor, where the tumor cells temporarily lose their epithelial characteristics and acquire mesenchymal characteristics instead [15]. Each radiomic feature harbors information regarding the tumor size, shape, or texture. Because texture-related features represent the heterogeneity of the tumor itself among various radiomic feature categories, radiologic and histopathologic characteristics can be complementary (Figure 3). The results in terms of feature selection add weight to this hypothesis. In this study, the final selected features were mostly texture features (85%), suggesting that these might be related to the tumor microenvironment profile, like EMT.

The prognostic relevance of TB status is well-established in colorectal cancers, which prompted the inclusion of this histopathologic characteristic in the recent WHO classification of colorectal cancer. Many studies showed that TB status was significantly associated with disease-specific survival in colorectal cancer [16,17,18,19]. In addition, T stage and lymph node metastasis were shown to be related to TB status in colorectal cancer [20]. Besides its prognostic relevance in colorectal cancer, it has been demonstrated that TB was associated with the prognosis of other cancer types, including esophageal, gastric, pancreatic, breast, and head and neck cancer in meta-analyses [21,22,23,24,25,26]. Our group revealed that integrated TB status could improve survival prediction models in cervical cancer [4]. Moreover, in our recent report, TB status was an independent prognostic factor for recurrence and nodal metastasis [5].

Although patients with early-stage cervical cancer treated with radical hysterectomy generally have a favorable outcome, ~20% of them experience recurrences. Several adverse prognostic factors for recurrence have been identified, such as tumor size, deep stromal invasion, LVI, positive resection margin, lymph node metastasis, and parametrial invasion. Among these factors, positive resection margin, lymph node metastasis and parametrial invasion are classified as high-risk factors, and adjuvant chemoradiotherapy is indicated [27]. Because tumor size, deep stromal invasion, and LVSI modestly increase the risk of recurrence, they are known as intermediate-risk factors [28,29,30]. When these three factors combined, the recurrence risk increased to 15–20%, which was similar to that in patients with high-risk factors. Although the Sedlis criteria has been the most frequently used in stratifying prognostic groups using these three factors, its prediction performance was disappointing [31]. Accordingly, there are efforts underway to find additional biomarkers in patients with intermediate-risk cervical cancer. Thus, radiomics and TB status, individually or comprehensively, are promising as new biomarkers. Preoperative information of TB status may make it possible to personalize treatment, including the extent of surgery and the addition of adjuvant therapy.

The final selected features in our prediction model were mostly texture features, which were derived from filtered images (Table 2). These findings were in contrast with those from the study of Li et al. [11], which showed that first-order statistics and shape features were selected for radiomic nomogram to predict LVI. In contrast, our results agreed with those of Hua et al., wherein the final selected features included three texture features and two first-order features. Note that all selected features in this study were features extracted after filtering. The features extracted from filtered images may reflect tumor characteristics not visible in the original images. The Laplacian of Gaussian filter can both sharpen and smoothen the original images, and it is commonly used for edge detection. A number of studies have reported that the features from Laplacian of Gaussian filters are related to tumor phenotype, gene expression, stage, and clinical outcome [32,33,34,35,36]. Wavelet filters can decompose the original image into low- and high-frequency components, and its incorporation in radiomics has improved prediction performance in other series [37,38]. For example, a combination of wavelet-filtered and unfiltered texture features improved the model performance for predicting pathologic complete remission status after neoadjuvant chemotherapy in a breast cancer study [37]. Taken together, the filtering method is a useful tool to capture the heterogeneity pattern in a spatial pixel distribution, allowing the improvement of its biologic relevance with radiomic features.

Despite the encouraging results, this study has some limitations. First, there may be concealed selection biases due to its retrospective nature. Second, its small number of patients may compromise its statistical reliability. Third, the prediction model was not validated through the external dataset, although an internal validation was performed. Therefore, the results should be cautiously scrutinized before its routine application in clinical practice. Despite these limitations, to the best of our knowledge, here we have the first report on an association between MRI radiomic features and TB status.

## 5. Conclusions

Preoperative MR-based radiomic features may be associated with TB status in patients with cervical cancer who were treated with radical hysterectomy. Therefore, MRI radiomic features have potential as practical imaging biomarkers predicting prognostic histologic characteristics, such as TB status.

## Figures and Tables

**Table 1:** Clinicopathologic characteristics of the 74 patients.

Characteristic	Training Set (*n* = 48)	Test Set (*n* = 26)	*p*-Value
Age (years)	49.1 ± 11.8	51.4 ± 10.1	0.4
FIGO stage (*n*, %)			0.517 ^†^
IB1	11 (22.9%)	3 (6.3%)	
IB2	12 (25.0%)	12 (25.0%)	
IB3	3 (6.3%)	1 (2.1%)	
IIA	1 (2.1%)	0 (0.0%)	
IIB	5 (10.4%)	3 (6.3%)	
IIIC1	16 (33.3%)	7 (14.6%)	
Histology (*n*, %)			0.580 ^†^
Squamous cell carcinoma	36 (75.0%)	18 (37.5%)	
Adenocarcinoma	8 (16.7%)	7 (14.6%)	
Adenosquamous carcinoma	4 (8.3%)	1 (2.1%)	
Tumor size (cm)	2.8 ± 1.6	2.6 ± 1.0	0.417
Lymphovascular invasion (*n*, %)	23 (47.9%)	11 (22.9%)	0.828
Deep stromal invasion (*n*, %)	33 (68.8%)	19 (39.6%)	0.903
Parametrial invasion (*n*, %)	12 (25.0%)	7 (14.6%)	>0.999
Lymph node metastasis (*n*, %)	16 (33.3%)	7 (14.6%)	0.76
Intratumor budding			>0.999
Intratumor budding counts	7.8 ± 9.9	7.7 ± 10.3	
Intratumoral budding (*n*, %)			
>4	27 (56.3%)	15 (31.3%)	
≤4	21 (43.8%)	11 (22.9%)	

Data are means ± standard deviations or numbers of patients with percentages in parentheses. Calculated by using the Student’s *t* test for continuous variables and the chi-square test for categoric variables, unless stated otherwise. ^†^ Calculated by using Fisher’s exact test.

**Table 2:** Selected radiomic features by univariate logistic regression analysis and LASSO regression in training dataset.

MR Sequence	Feature Name
T1	log.sigma.1.0.mm.3D_glszm_ZonePercentage log.sigma.3.0.mm.3D_ngtdm_Contrast wavelet.LLH_gldm_LargeDependenceEmphasis wavelet.LHH_firstorder_Skewness wavelet.HLL_glszm_SizeZoneNonUniformityNormalized wavelet.HLH_gldm_LargeDependenceHighGrayLevelEmphasis wavelet.HHL_gldm_DependenceVariance. wavelet.LLL_glcm_InverseVariance.
T2	log.sigma.3.0.mm.3D_ glcm_Imc2 wavelet.LLH_glszm_HighGrayLevelZoneEmphasis wavelet.LLH_glszm_LowGrayLevelZoneEmphasis wavelet.LLH_glszm_SmallAreaHighGrayLevelEmphasis wavelet.LHL_gldm_HighGrayLevelEmphasis wavelet.LHL_gldm_LowGrayLevelEmphasis wavelet.LHL_glrlm_HighGrayLevelRunEmphasis wavelet.LHL_glrlm_LowGrayLevelRunEmphasis wavelet.LHL_glszm_GrayLevelNonUniformityNormalized wavelet.LHH_firstorder_Kurtosis wavelet.HLH_glszm_ZoneEntropy wavelet.LLL_firstorder_Minimum

LoG, Laplacian of Gaussian filter; gldm, gray level dependence matrix; glszm, gray level size zone matrix; glcm, gray level co-occurrence matrix; glrlm, gray level run length matrix.

**Table 3:** Performance of machine learning classifiers for predicting tumor budding status in test dataset.

Classifier	AUC (95% CI)	Accuracy (95% CI)	Sensitivity	Specificity	PPV	NPV
LR	0.742 (0.572–0.907)	0.769 (0.564–0.910)	0.857	0.737	0.546	0.933
RF	0.782 (0.528–0.884)	0.731 (0.522–0.884)	0.750	0.722	0.546	0.867
SVM	0.849 (0.740–1.000)	0.885 (0.699–0.976)	0.900	0.875	0.818	0.933
NN	0.891 (0.768–1.000)	0.731 (0.522–0.884)	0.667	0.786	0.727	0.733

AUC, area under curve; CI, confidence interval; PPV, positive predictive value; NPV, negative predictive value; LR, logistic regression; RF, random forest; SVM, support vector machine; NN, neural network.

## Data Availability

The datasets generated and/or analyzed during the current study are not publicly available due to the privacy protection policy of personal medical information at our institution, but are available from the corresponding author on reasonable request.

## Supplementary Materials

The following are available online at https://www.mdpi.com/article/10.3390/cancers13205140/s1, Figure S1: Flowchart of patient inclusion, Figure S2: Feature selection performed by least absolute shrinkage and selection operator regularization.
