# Supplementary figures and images for: Predicting Tumor Budding Status in Cervical Cancer Using MRI Radiomics: Linking Imaging Biomarkers to Histologic Characteristics
Source: Cancers (Basel). 2021 Oct 14;13(20):5140. doi: 10.3390/cancers13205140 (PMC8534175; doi:10.3390/cancers13205140)

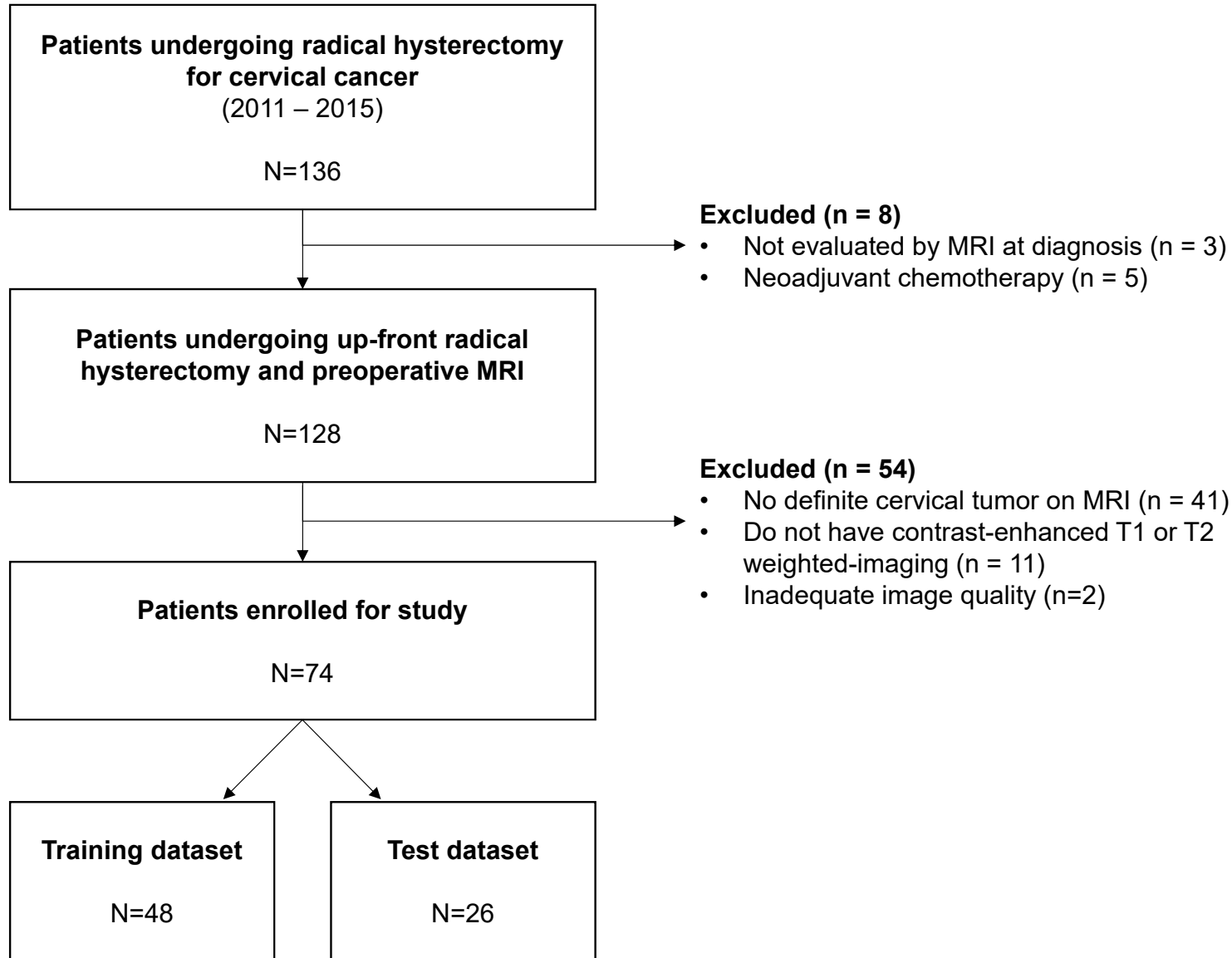

Supplement: Supplementary file 1 [file cancers-13-05140-s001.zip › Figure S1.pdf]

(a)

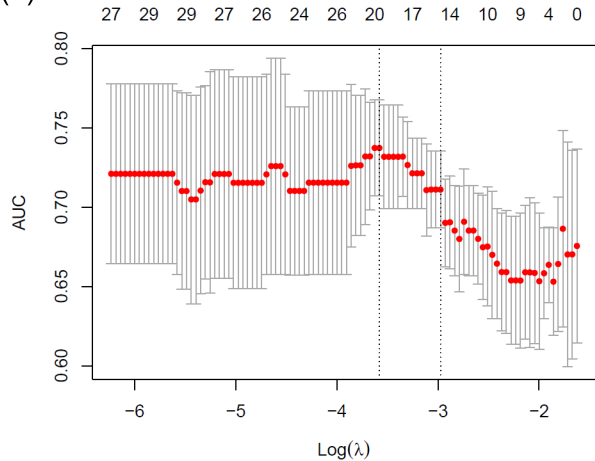

(b)

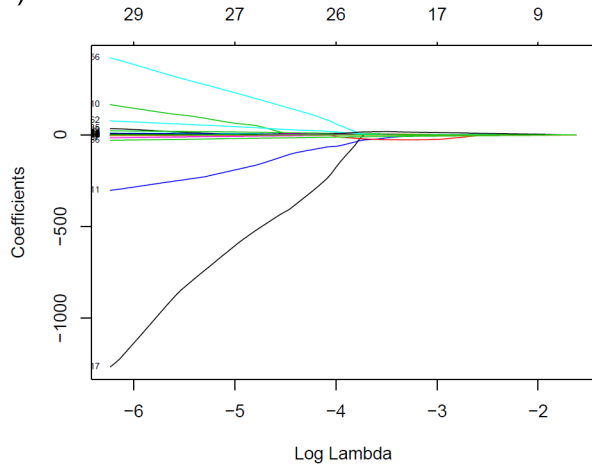

Supplement: Supplementary file 1 [file cancers-13-05140-s001.zip › Figure S2.pdf]
